# Reward of Physical Labor

**Published:** 1856-08

**Authors:** 


### REWARD OF PHYSICAL LABOR.

At the funeral of a clergyman who died with his harness on, at the age of eighty-eight years, it was said of him: "He was favored with a robust and healthy constitution. On his father's farm he acquired the habit and love of agricultural labor, which he retained through life, and which contributed so eminently to the health and vigor, which, with scarcely any interruption, he enjoyed all his days."

We believe that the Church commits an error in putting young men into the ministry so early. If the Divine Author of our religion worked at the trade of a carpenter until he began to be about thirty years of age, we see no sufficient reason why men less divine, and so immeasurably less gifted, should hurry into it at an earlier period, with all their inexperience of men and things; that very inexperience which has led many a talented young clergyman into the commission of mistakes, which have colored a subsequent lifetime; mistakes, which have made life a failure.

We are not sure that a five years' course of working with one's hands for daily bread would not, in the long run, be productive of incalculable benefits to the Church and to the world.

*First.* It would raise up a ministry of robust health, capable of performing in one year more real hard work in the field of the world, than would a score of theological fledgelings of the present day.

*Second.* It would give a ministry who, knowing something of human nature, could sympathize with its sorrows, could compassionate its weaknesses, and could, having been tempted as we are, be touched with a feeling of our infirmities, could weep with those who weep, and rejoice with those who rejoiced.

